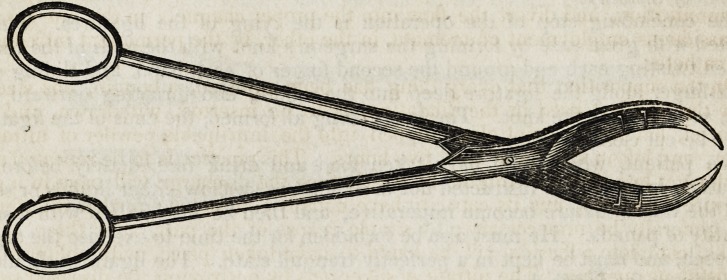# Surgery

**Published:** 1836-01

**Authors:** 


### SURGERY.

*Observations on Staphyloraphy, or Palate-Suture, and on Extirpation of the Tonsils.* By N. R. SMITH, M. D. Professor of Surgery in the University of Maryland.

[WHILE appreciating the ingenuity and adroitness displayed by Dr. Smith in the adaptation of mechanical means to the performance of surgical operations, we must be permitted to warn our younger surgical brethren, that no mechanical contrivances can make amends for, much less supersede the necessity of, cultivating a thorough anatomical knowledge of parts, and a most familiar acquaintance with the knife. It is needless to say, that nothing but frequent practice, as well on the living as the dead body, can give this knowledge and this skill. Improved instruments may make real knowledge more effective, but can never be its substitute.]

*Staphyloraphy.* Having been twice called upon, (says Dr. Smith,) for the performance of this operation by persons from abroad, who could not remain until I procured the instruments which I then had not, I have been under the necessity of using more simple means, and I have succeeded with so much facility that I am confident I shall never employ any other.

The only instrument, besides those found in the ordinary pocket-case, at all necessary for the execution of staphyloraphy, is the needle represented in the accompanying cuts; and it is so simple an instrument that I made the first with my own hands in a few minutes. It is a needle having a permanent handle, its point a little broader than common, and bent from the point into a semicircle, the radius of which is about half an inch. Instead of having an eye near the point, as commonly, this form of needle has merely a notch upon one of its edges. This notch passes obliquely inward and backward from the point, reaching the centre of the blade of the needle. The blade, just anterior to the notch, is broader than it is behind it, so that when the needle is thrust through the soft parts, the posterior angle of the notch passes through without impediment. This needle is armed by simply laying the ligature into the notch—the thread being of such size that it accurately fits it, and waxed so as to make it slightly adhere. It is then employed in the following manner: One end of a long ligature is laid into the notch of the needle. The patient being placed in a convenient posture, and the jaws kept widely expanded by the insertion of a cork between the molar teeth, the surgeon introduces the needle into the mouth; carries its curved portion behind the cleft palate, directing its point behind the middle of the uvula. He then brings forward the instrument, so as to thrust its point from behind forward, through the body of the uvula. If the uvula does not furnish resistance enough to enable him to do this with ease, he may seize its point at the moment with a pair of slender forceps. As soon as the point of the needle is brought fairly through, and pushed forward so far that the thread in the notch appears, the operator may withdraw the needle from the part with a slow motion, and the thread will generally slip with ease from the notch, and be left in the wound. Or that this may occur with the more certainty, the thread, when it appears through the wound, may be seized with forceps, or small hook, and disengaged from the notch.

In a manner precisely similar, another ligature is to be passed through the border of the cleft palate, about half an inch above the former. In many cases a third ligature may be inserted at the same distance above the last; but it is better that this should not be attempted when it is manifest that very strong traction of this last ligature will be necessary to bring together the opposite margins. The ligature would then give rise to much irritation, and not only fail to effect union where it is applied, but embarrass and perhaps defeat the process of adhesion in the parts included in the other ligatures.

The next step is the paring of the border of the velum. This I have accomplished with great ease by seizing both ends of the ligature, with which the uvula is transfixed, and with them pulling the uvula forward. This will render this portion of the velum nearly horizontal, and the operator may easily pare its border with scissors, either straight or having a slight lateral curvature. Or the surgeon may use a straight probe-pointed bistoury for this purpose; but the ligature must then be neld by an assistant, and the surgeon must seize the inner border of the uvula with slender forceps. Care is to be taken, in accomplishing this step, not to cut the upper ligatures; and this may be avoided without difficulty by thrusting them well back behind the velum.

The surgeon next proceeds to pass the other extremities of his ligatures through the opposite portion of the velum. This is effected in precisely the manner described above. To pare the border of the velum on this side, however, it is necessary to proceed a little differently. The loop of the lower ligature, which now passes behind the velum from one side to the other, is to be hooked forward and drawn out of the mouth. This loop is then to be held by the operator, together with the end of the ligature on the side upon which he is now operating. Still greater care is here necessary to avoid cuttiug the ligatures above, while using the scissors or bistoury.

The concluding step of the operation is the tying of the ligatures. This is effected with great ease by forming the surgeon's knot with the ends of the thread —then twisting each end around the second finger of each hand, and sliding each index-finger along the ligature deep into the mouth, and thrusting outward with each, so as to close the knot. The knots being all formed, the ends of the ligatures are to be cut close to them.

The patient, who should have taken food and drink immediately before the operation, is now to be instructed not to attempt to swallow either liquids or solids until the calls of nature become imperative, and then he may be fed with a small quantity of panada. He must also be forbidden for the time to exercise the organs of speech, and must be kept in a perfectly tranquil state. The ligatures should be cut away on the fourth day.

When the fissure traverses not only the soft palate, but also the bony portion, as is usually the case, the operator of course does not expect to close the anterior portion of the fissure. If, however, he succeeds in effecting pretty firm union of even a portion of the velum palati, near the uvula, he has accomplished an important object, and has achieved the principal end of the operation. It is now easy, by the employment of a suitable obturator, to close the anterior portion of the opening.

*Extirpation of the Tonsils.*—The extirpation of enlarged tonsils is by no means an operation of very formidable character, but it is one which is often necessary, and which is generally accomplished with some difficulty. Hence the multitude and variety of instruments which have been devised for effecting it. That no instrument for this purpose has yet been employed free from important imperfections is obvious, from the fact, that almost every medical journal furnishes some new device for this purpose, intended to supply deficiencies.

The ligature, for the removal of enlarged tonsils, has been by prudent surgeons almost entirely abandoned. The bug-bear, hemorrhage, which has frightened so many into the continued use of it, has lost its terrors. The fact is, that it is not necessary ever to cut away the entire base of the tonsil; if it be only cut through in the centre, so that one half is removed, it will generally be found sufficient; for, in the first place, suppuration will waste a portion of the remainder, and then the cicatrix subsequently forming will repress it. I rarely attempt to cut away more than two thirds of the tumor, and this portion of it is not very vascular, nor has it much sensibility. Those appalling instances of hemorrhage of which we read, have no doubt arisen from the fact that the incision has been carried too deeply into the base of the tumour, wounding vessels which do not wholly pertain to the tonsil. This more frequently happens when the tumour is seized with forceps, and dragged out from its recess at the moment that it is excised. The ligature is a tedious, painful, and often inefficacious remedy. I have wholly abandoned its use for some years.

The knife is not easily applied, and requires to be aided by forceps. The common scissors do not seize the tumour with such a hold as to complete its division at one stroke, as is desirable. Whatever instrument is employed, the excised portion of the tonsil is almost always left in the throat, and it becomes necessary to remove it with the forceps, or the patient swallows it.

A few days since I undertook the extirpation of very much enlarged tonsils in the throat of a small child. I had at hand almost every variety of instrument used for this purpose, as I anticipated considerable difficulty. I tried nearly all in succession, without being able to effect my object, but finally succeeded clumsily with a pair of large probe-pointed scissors, first having seized the tonsil with slender, toothed forceps. Whilst the difficulties of the case were fresh in my mind, I resolved to provide myself with something which should obviate similar embarrassment. I had found that the scissors which I used would have answered extremely well had they had a regular lateral curve, and had they been provided with any appendage to prevent the tonsil from slipping from the grasp of the blades in the direction of their points. Something also, it occurred to me, might be appended, that should seize the severed tonsil at the moment of its division.

With these objects in view, I put into the hands of the instrument maker a model of the instrument here represented. It will be seen that it is a pair of scissors, the blades of which have a lateral curve, and each a hawk-bill curve toward the other, so that when the blades are shut, the points pass by each other to some extent. When any thing of the size of a diseased tonsil is seized by these scissors, the moment the middle part of the blades begins to press it, the points meet and begin to pass each other, so that the tumour cannot possibly escape. To the side of each blade there are attached two small steel points, which are bent toward the edges of the blades, so that when the scissors are completely closed, the tonsil exterior to the place of the incision will be seized and held by the points, and brought away from the fauces when the instrument is withdrawn. The blades should be about an inch and three-fourths in length from hinge to point.

In using this instrument, forceps for seizing the tonsil are unnecessary, nor need we employ any thing for the purpose of keeping the mouth open, as when the scissors are introduced the mouth cannot be closed. The lateral curvature of the blades enables us to press them as deeply as we please into the recess in which the tonsil is lodged.

These scissors will be found equally useful for the extirpation of the uvula, and for the removal of hemorrhoids.—*American Archives of Med. Sciences.* No. 2. *November* 1834.

---

*On the Cure of Fistulæ and Ulcers.* By Dr. Gottlieb Cramer.

A point of primary importance is the opening up and uniform dilatation of the sinus, whereby the diagnosis is certified, the issue of the secreted matter favoured, and the needful application of medicinal substances facilitated. To fulfil the former indication, a wax bougie, of such a caliber as permits its easy entrance, is to be frequently introduced, until it passes readily to the very extremity of the fistula; it should never be attempted to force a passage when it meets with opposition, but the point be allowed to remain in contact with the obstacle until that is overcome. Having then arrived at the bottom of the now freely permeable sinus, it will be necessary to proceed with its gradual dilatation. For this purpose, the bougie is to be allowed to remain in the canal a couple of hours daily, gradually increasing the thickness of the bougie until the requisite dilatation has been effected, and the fistula transformed into a tolerably equiform cylindrical cavity, from its external orifice down to its termination. A fortnight will in general suffice to bring about this end; not only will the secretion then be improved in quality, but its egress promoted and its accumulation prevented. The redness, pain, and swelling in the surrounding parts will have diminished, and the secerning membrane will have acquired tone.

Having thus fulfilled the proposed indications, it will be proper to resort to means which will ensure the cicatrization and permanent closure of the canal, by gradually circumscribing the morbid secreting surface, and causing it to heal from below upwards. Should the suppurative process be maintained by the pressure of a foreign body or other irritant, as a portion of carious or necrosed bone, that must be removed. Should it be kept up, in the case of its terminating in one of the natural canals, from the contents flowing along it, as is found to hold good in the instance of stercoraceous or urinary fistulæ, we must first of all seek to stop up the communication, and divert the flow into the proper channel; in the one case, by the assiduous employment of enemata, in the other, by the permanent introduction of a catheter.

On the supposition that every thing has been adjusted, a bougie, one size less than the one last used in the dilatation, is to be inserted, withdrawn, and then replaced, having its point alone dipped into the impalpable powder of nitrate of silver, and left in situ for at least two hours. This practice is to be repeated every day for not less than a fortnight, by which time the discharge will have in a great measure decreased, and its capacity have notably contracted; further interference will generally be unnecessary; by simple repose of the part, after three or four weeks, complete adhesion will be found to have taken place, with obliteration of the fistulous opening. The nitrate of silver acts by destroying the diseased surface with which it is brought into contact, a slough forms, below which a new and different inflammatory action is set up. The slough is at length eliminated, the dilated capillaries contract, a healthy suppurating surface, like that from a recent wound, is substituted for the former, tending rapidly to cicatrize and heal up.

Several cases are adduced in support of the efficacy of the method, which it is asserted has been advantageously extended to the cure of chronic abscesses, where there is much atony present.

The plan Dr. C. recommends for the treatment of ulcers, consists in dipping the piece of lint imbued with the discharge in the pulverized nitrate of silver, and re-applying it on the sore. This he repeats every day, or every other day, and by enforcing a quiescent state of the member, he mentions his having succeeded in healing the most obstinate ulcers in a period of six or eight weeks. He has likewise employed the same powder with advantage to the granular conjunctiva.

*Heidelberger Klinische Annalen*, Bd. x. H. 1. pp. 71—139.

---

*On permanent Retraction of the Fingers.* By M. GOYRAUD, D.M.

DUPUYTREN described in his lectures a permanent contraction of the fingers to which coachmen and others were subject, whose occupation required the constant flexure of the fingers. Extension was prevented by strong bands running beneath the skin, from some part of the palm to the fingers: and from several dissections, Dupuytren decided that these cords were produced by a permanent contraction of portions of the palmar aponeurosis. M. Goyraud, as well as M. Velpeau, from examinations, object to this explanation, for the digital slips of the palmar aponeurosis terminate in and are fixed to the base and sides of the root of each finger, whilst the diseased band is situated in the middle of the finger, and is often prolonged to the third phalanx. They both therefore agree in referring this contraction to a transformation into a fibrous band of a part of the subcutaneous tissue in front of the phalanges. M. Sanson thinks that this is the common cause, and that contraction of the palmar aponeurosis is an exception. The treatment recommended by M. Goyraud, is to make a longitudinal incision through the skin over each band when extended, to separate the lips of the wound, to detach the fibrous cords by dissection, and to cut across these cords thus isolated. If these cords in front of the fingers send processes to the first phalanges, and then pass on to be inserted into the second, they should be cut above and below these processes. If the section of these cords leaves in the wound partially detached shreds, they should be cut out. The fingers should be completely extended and fixed, and the wound healed by the first intention.

Sir A. Cooper recommends a small bistoury to be passed under the cord, which should then be divided so as not to cut through the skin; but this is only applicable to those rare cases where the cord is not adherent to the skin. Dupuytren recommends a transverse section of the cords and of the skin. M. Goyraud considers his own method preferable, as his longitudinal incisions will heal more rapidly than transverse, which must necessarily suppurate, and therefore are less likely to be attended with inflammation, which might injure the parts beneath, whilst the cicatrix would be linear, instead of broad and adherent.

*Gazette Médicale*, No. 31, 1 Août, and No. 32, 8 Août, 1835.

---

*On the Treatment of Ozœna and other Diseases of the Organ of Smell.* By Dr. James Heron, President of the Medical Society of Orange County, in the State of New York.

Ozœna, or the fœtid, purulent discharge from the nostril, is ordinarily termed catarrh; in its more advanced state, cancer of the nostril or throat, as it occupies principally the one or the other situation.

I have in my recollection an aged friend, who frequently complained to me of what he called catarrh, and that he was often in the night compelled to spring up in his bed by a sense of suffocation, caused by the falling of large quantities of matter from the back part of the nostril into the throat, while he slept. He was found dead in his bed one morning, at a time when he was enjoying his ordinary health, and this was believed to be the most rational cause of death.

Another died the past season by starvation and distress, occasioned by ulceration extending to and occupying the pharynx, so that for a considerable time before his death he could not swallow a particle of nourishment. The termination of this case was called cancer of the throat: and the patient had had for a number of years previous a very fœtid discharge from the nostril, accompanied with pain and soreness in the region of the frontal sinus.

And I now know a member of our profession, aged about 30 years, whom this disease has doomed and marked for destruction at no distant day. Ulceration has entirely destroyed the soft part of the right ala of the nose, the inferior spongiosum, the septum narium, a considerable part of the tonsil, palate and pharynx. The stench from the ulceration is so great that no person can comfortably be near him, although he endeavours to correct it by the most powerful aromatics;—deglutition is so difficult, that he scarce ever swallows anything but liquids, and these frequently pass out at the nostril. He has not for more than a year lain on his back, as the matter then passes into the fauces and endangers suffocation. For this reason he scarcely ever lies down at all, but sleeps in an easy chair with his head inclined forward on a support.

Such had been the inefficiency of the treatment of ozœna but a few years ago, that men so eminent as Boyer and Dr. Physick were induced to pronounce it absolutely incurable in its advanced stage. About three years since, I had a case of this disease which baffled all my resources—and when I sought for light in relation to it, by conversing with my medical friends, I was disappointed, until my attention was directed to some cases recorded in the American Journal of Medical Sciences, published in Philadelphia, which were successfully treated by injecting chloride of lime in solution to the part affected. Since then I have used in all affections of this kind the diluted liquid chloride of soda, and the chloride of lime in solution, indiscriminately, and with equal success; and so confident am I of their efficacy, that I believe no ordinary case of ozœna will resist their proper use. I have also found that much benefit will be derived from passing the smoke of Spanish tobacco through the diseased nostril several times a day, and that the application of olive oil to the internal surface of the nostril is necessary to prevent the excoriations which the ichorous discharge is apt to occasion, and also to prevent the hard and dry incrustations into which the matter in passing is apt to collect.

In the application of injections to the upper and posterior part of the nostril, some management is necessary. The injection should be made while the head is inclined forward and downward; at the same time, by inhaling the breath through the nostril the injected fluid will be brought into contact with the diseased surface, where it may be retained as long as is necessary.

I have never been able to use the injection of chloride of lime of the strength it was used by Dr. Horner, (viz. a tea-spoonful to a wine-glassful of water) : my experience teaches me that applications made here (as to the eye) are better when they are moderately sharp, than when they are too strong. The sensation produced on the olfactory nerve by the application of the chloride of lime or soda, is of that pungent kind frequently the consequence of a free use of mustard or horseradish—and any one can conceive how distressing it would be to use the injection beyond a bearable strength. These applications excite a free discharge of the natural healthy mucus of the follicles of the membrane of the nose, and effectually correct the fœtor of the discharge; in which particular they exceed every other application I have known to be made; and which would be sufficient inducements, if none other offered, to persevere in their use. They should be used twice a day, i. e. morning and evening.—*United States Med. and Surg. Journal for October* 1835.

---

*New Treatment of Blenorrhagia in Women.* By P. RICORD, Surgeon of the Venereal Hospital, Paris.

[M. RICORD, in consequence of the difficulty of curing chronic discharges from the vagina and uterus at the Venereal Hospital, was induced to use strong injections of nitrate of silver, such as had been recommended in this country in the gonorrhœa of men, and also the solid nitrate. The following is the result of his trials.]

He used at first injections composed of ten grains of nitrate of silver to an ounce of water, but as the first trials were not followed by benefit, he conjoined with injections, compresses wet with the solution, which were left in the vagina, directing their removal if they produced pain. Some retained them two or three hours, when they felt some heat; but others twenty-four hours. On examining the vagina with a speculum the next morning, the mucous membrane was covered with a dark pellicle; and when this became detached on the following days, the mucous membrane beneath it appeared pale, without any sign of inflammation or ulceration. In some cases this stopped the discharge for two or three days, when it returned in less quantity: in others the secretion was at first increased, but gradually diminished. In all cases the injection was repeated at four or five days' interval, and in some no benefit was derived. Encouraged by a certain number of successful cures of discharge from the vagina, M. Ricord next tried injecting the uterus, with a syringe expressly adapted for this purpose. This was followed by heat in the hypogastric region, by some nervous movements, and in three cases by a complete cure. The injection in the first case brought on the menses eight days before their time, and was followed by slight menorrhagia, lasting a fortnight, after which the purulent discharge ceased. In the second, three injections at eight days' interval were necessary: the menstrual secretion was increased. In the third, where there was amenorrhœa of six months' standing, the menses followed the second injection. Fear of bad consequences following this mode of injection, led M. Ricord to substitute another. A speculum is introduced, and the os uteri touched lightly with nitrate of silver, so as to whiten the mucous membrane; the speculum is then gradually withdrawn, and, as the vagina appears at its extremity, it is likewise touched. Many times daily during the next four or five days an injection is used, consisting of one ounce of acetate of lead, dissolved in two pounds of water.

The speculum is again introduced at the end of this period, and those parts which do not bear the marks of the nitrate of silver are again touched. Two, three, four, or five applications may be necessary, at four or five days' interval. Superficial cauterization may be made without the speculum, by introducing a long caustic holder to the neck of the uterus, and then withdrawing it gently and turning it. This has been followed with considerable success; and is not only useful in chronic cases with or without alteration of the tissue of the mucous membrane, but in the acute stage, either at first or after a certain duration of the symptoms. The inflammatory symptoms were never augmented. For uterine blenorrhagia or catarrh, the caustic has been introduced into the cavity of the neck of the uterus with equally favourable results and without accidents; and in such cases it has often acted as an excellent emmenagogue, and might be used as such.

[The introduction of strong injections or of the actual nitrate into the cavity of the uterus, is too hazardous an experiment to be recommended. From physiological researches we know that the semen passes into the Fallopian tubes, a fact which certainly should put us on our guard against forcing in irritating fluids in such a way that they might pass even into the peritoneal sac. The strongly rooted aversion among females in this country to such a remedy as the introduction of the solid caustic would exclude it from emmenagogues, and confine its use even in vaginal discharges to our sick hospitals.]

*Bulletin Géneral de Thérapeutique, Juin,* 1835.

## Figures and Tables

**Figure f1:**
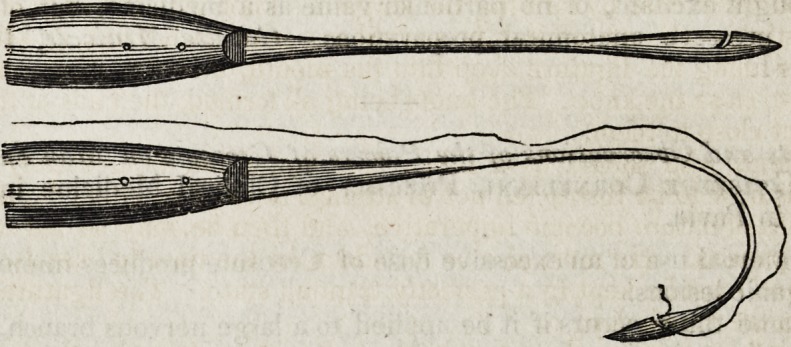


**Figure f2:**